# Efficacy and cost-utility of the eHealth self-management application 'Oncokompas', helping partners of patients with incurable cancer to identify their unmet supportive care needs and to take actions to meet their needs: a study protocol of a randomized controlled trial

**DOI:** 10.1186/s13063-019-4037-5

**Published:** 2020-01-31

**Authors:** Anouk S. Schuit, Karen Holtmaat, Nienke Hooghiemstra, Femke Jansen, Birgit I. Lissenberg-Witte, Veerle M. H. Coupé, Irma M. Verdonck-de Leeuw

**Affiliations:** 1 Department of Clinical, Neuro and Developmental Psychology, Faculty of Behavioural and Movement Sciences, Amsterdam Public Health Research Institute, Vrije Universiteit Amsterdam, van der Boechorststraat 7, 1081 BT Amsterdam, The Netherlands; 2 Cancer Center Amsterdam (CCA), Amsterdam Public Health Research Institute, Amsterdam, The Netherlands; 3 Otolaryngology – Head and Neck Surgery, Cancer Center Amsterdam, Amsterdam UMC, Vrije Universiteit Amsterdam, De Boelelaan 1117, 1081 HV Amsterdam, The Netherlands; 4 Department of Epidemiology and Biostatistics, Amsterdam UMC, Vrije Universiteit Amsterdam, De Boelelaan 1117, 1081 HV Amsterdam, The Netherlands

**Keywords:** Incurable cancer, Caregiving, Partners, eHealth, Self-management, Caregiver burden

## Abstract

**Background:**

Incurable cancer does not only affect patients, it also affects the lives of their partners. Many partners take on caregiving responsibilities. The burden of these caregiving tasks are often associated with physical, psychological, and social difficulties and many partners have unmet supportive care needs. Oncokompas is an eHealth self-management application to support partners in finding and obtaining optimal supportive care, tailored to their quality of life and personal preferences. A randomized controlled trial will be carried out to determine the efficacy and cost-utility of Oncokompas.

**Methods:**

A total of 136 adult partners of patients with incurable cancer will be included. Partners will be randomly assigned to the intervention group, which directly gets access to Oncokompas, or the waiting-list control group, which gets access to Oncokompas after three months. The primary outcome measure is caregiver burden. Secondary outcome measures comprise self-efficacy, health-related quality of life, and costs. Measures will be assessed at baseline, two weeks after randomization, and three months after the baseline measurement.

**Discussion:**

This study will result in evidence on the efficacy and cost-utility of Oncokompas among partners of patients with incurable cancer, which might lead to implementation of Oncokompas as a health service for partners of patients with incurable cancer.

**Trial registration:**

Netherlands Trial Register, NTR 7636. Registered on 23 November 2018.

## Background

It is well known that cancer does not only affect patients; the disease also has a considerable impact on the lives of their partners [1, 2]. Partners of patients with incurable cancer often help with personal care and provide practical and emotional support to patients [3, 4]. It is not uncommon that they perform caregiving tasks they are not trained for (e.g. the management of medication and symptoms). Partners may feel overwhelmed by these tasks. They often also consider their own problems as less important than those of the patient [5, 6]. Since cancer increasingly becomes a chronic illness, partners of patients with cancer are challenged to be involved in the management of the patient’s care and quality of life for an increasing extent of time, while also maintaining their own wellbeing [7].

Although caring for a loved one can be rewarding [8], informal caregiving responsibilities are also associated with physical, psychological, and social difficulties [1, 4, 9–11]. Frequently reported symptoms among caregivers are sleeping problems, fatigue, and psychological distress [12–14]. Many partners have to give up (part of) their normal daily activities due to their caregiving tasks, for example their work or social activities [1, 15]. Partners may experience high burden levels related to their responsibilities and the impact of the caregiving on their daily lives [1, 5, 16]. Caregiver burden is defined as “the extent to which caregivers perceive that caregiving has an adverse effect on their emotional, social, financial, physical, and spiritual functioning” [17]. Studies have shown that these adverse effects negatively influence the quality of life of partners [1, 2, 9–14, 18–20].

Many partners do not know where to go for advice and guidance or do not have time to seek help [13, 21–23]. Therefore, there is a growing interest in self-management interventions and eHealth applications as ways to improve (the early access to) supportive care targeting partners of patients with incurable cancer [7, 24, 25].

The eHealth self-management application Oncokompas has been developed to support patients and partners of patients with incurable cancer in finding and obtaining optimal supportive care. Oncokompas helps them to monitor their quality of life using patient-reported outcome measures (PROMs), followed by automatically generated tailored feedback, self-care advice, and advice on supportive care services. The content of the version of Oncokompas for partners is focused on self-care of the partner and targets the partner alone instead of the couple (i.e. patient and partner together), for example to inform and advise partners about their shifting roles and responsibilities, their relationship, financial resources, and their work situation. The application is tailored to the partner’s health status and personal preferences. There is a dedicated version of Oncokompas available for patients treated with curative intent [26–28] and for patients with incurable cancer [29]. The aim of this randomized controlled trial (RCT) is to determine the efficacy of Oncokompas as a self-management instrument on caregiver burden, general self-efficacy, and health-related quality of life among partners of patients with incurable cancer and to assess its cost-utility.

## Methods/Design

### Study design

A prospective RCT with two parallel groups will be conducted to determine the efficacy and cost-utility of Oncokompas among partners of patients with incurable cancer.

**Fig. 1:**
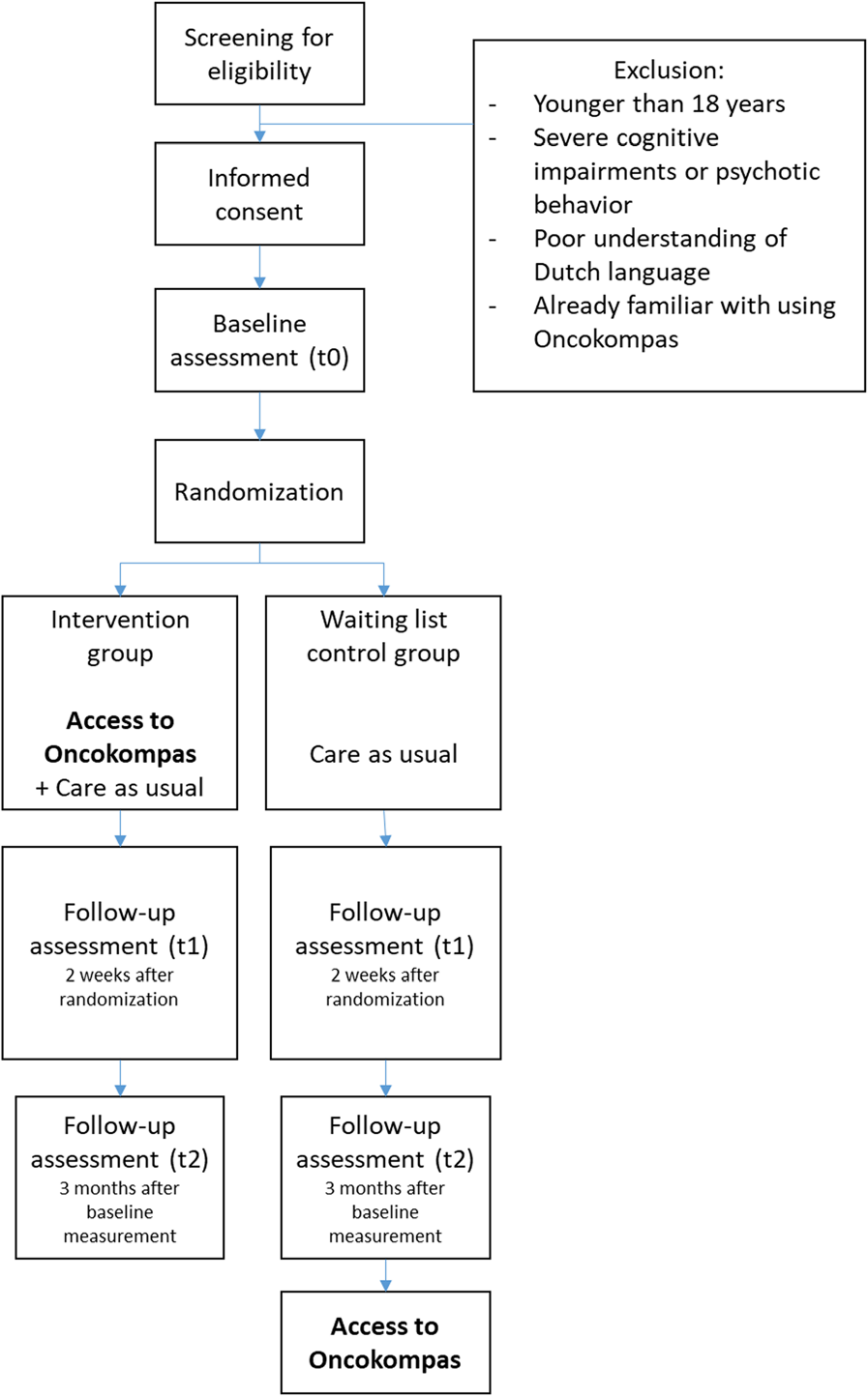
Flow diagram of the randomized controlled trial

**Fig. 2:**
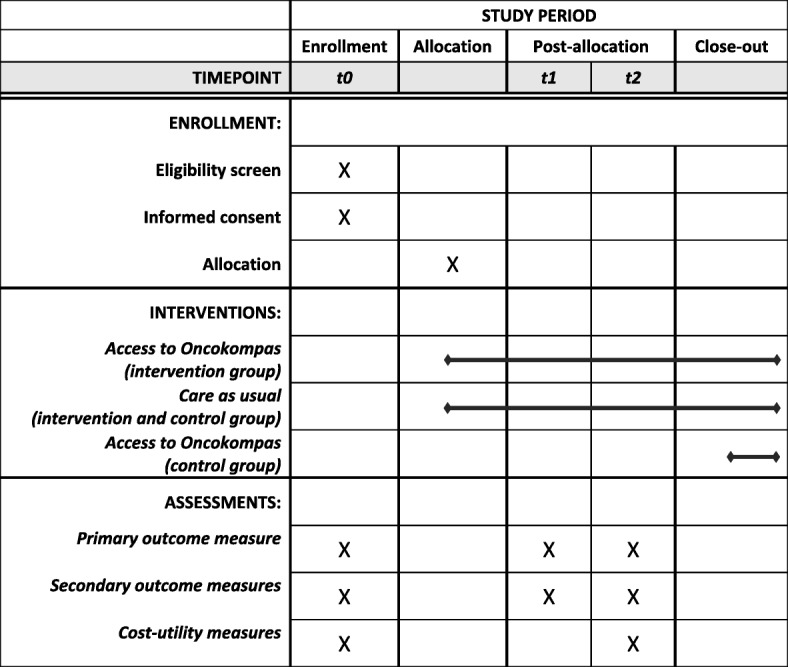
The schedule of enrollment, intervention, and assessments of the randomized controlled trial (according to SPIRIT)

Partners will be randomly assigned to the intervention group or the waiting-list control group. Partners in the intervention group will get direct access to Oncokompas, while partners in the control group will get access to the intervention three months after the baseline measurement (i.e. after completion of the last questionnaire). Partners will receive three questionnaires: at the time of inclusion (t0), two weeks after randomization (t1), and three months after the baseline measurement (t2). Figure 1 shows the flow diagram of the RCT. Figure 2 shows the schedule of enrollment, intervention, and assessments (according to the Standard Protocol Items: Recommendations for Intervention Trials (SPIRIT), see Additional file 1).

This study is approved by the VUmc Medical Ethical Committee (registration number 2018.517). All respondents will provide written informed consent before inclusion and will be informed that participation is voluntary. Partners can withdraw from the study at any time without any consequences.

### Study population

#### Inclusion and exclusion criteria

In this study, partners of patients with incurable cancer will be included. Partners are included when they are aged ≥ 18 years and have access to an e-mail address. Partners are excluded when they have severe cognitive impairments or psychotic behavior, or when they have a poor understanding of the Dutch language (and thereby are not able to complete a questionnaire in Dutch). They will also be excluded when they already used Oncokompas earlier in life (e.g. if they have had cancer themselves) or when their partner with cancer participates in the Oncokompas RCT, which is currently conducted among patients with incurable cancer [29].

### Study procedures

In this study, a multi-component recruitment strategy is followed. Partners will be recruited through: (1) (online) recruitment materials, (2) healthcare professionals, and 3) direct contact with the researcher. Table 1 gives an overview of the different recruitment strategies used within this study.

**Table 1:** Overview of the different recruitment strategies

Recruitment strategy	Recruitment channel
Recruitment through (online) recruitment materials	Online: • Online advertising on websites and online newsletters • Social Printed: • Advertisements in newspapers and magazines • Leaflets and posters in offices of healthcare professionals Recruitment through: • Relevant organizations targeting informal caregivers or relatives of (cancer) patients • Cancer patient organizations • Walk-in consultation services • Hospitals • Psycho-oncological care centers
Recruitment through a healthcare professional	• Healthcare professionals (e.g. psychologists, rehabilitation centers, general practitioners, physiotherapists, nurse practitioners)
Recruitment through face-to-face contact with the researcher	• Events targeting partners of patients with incurable cancer

#### Recruitment through (online) recruitment materials

Several recruitment materials have been developed to recruit partners through online channels. The contact details of the researcher and URL of the website of Oncokompas (www.oncokompas.nl) are mentioned in all recruitment materials. On the Oncokompas website, partners can find more information about Oncokompas and the study, such as how they can apply to participate. When partners are interested in participating in the study, they can fill in an online contact form on the website.

#### Recruitment through a healthcare professional

Partners eligible to participate will also be approached through healthcare professionals. When a partner is interested in participating in the study, the researcher will contact the partner by phone to further inform him or her about the study.

#### Recruitment through direct contact with the researcher

Partners will also be informed about the study on events targeting relatives of patients with incurable cancer. If interested, they will receive an information letter about the study.

To summarize, many organizations throughout the Netherlands will be involved in the study by informing and referring partners of patients to the website of Oncokompas (or directly to the research team); all other actions regarding the study are carried out by the research team of the Vrije Universiteit Amsterdam, the Netherlands. Therefore this study is marked as a monocenter study.

#### Partners who want to participate

Partners meeting the inclusion and exclusion criteria will receive an information package by post (consisting of an information letter, an informed consent form, and a reply envelope). If partners want to participate in the study, they are asked to return the signed informed consent form using the reply envelope. After the coordinating researcher has received the signed informed consent form, this researcher will send partners a link to the online baseline questionnaire by e-mail. After completion of the first questionnaire, partners will be randomized into the intervention group or the control group. Partners randomized to the intervention group will receive an invitation e-mail for Oncokompas to activate their personal account. Partners randomized to the control group will receive an e-mail to activate their account after completion of the last questionnaire (t2).

#### Randomization

Randomization takes place in a 1:1 ratio. Block randomization will be used to randomly assign partners to the intervention group or the control group. Block size varies between 4–8. Random allocation software (i.e. Sealed Envelope) is used by a researcher not involved in the study to create the randomization scheme. This researcher also carries out the allocation process during the study and notifies the coordinating researcher of the study of the outcome of the allocation. The coordinating researcher will send partners the invitations to activate their Oncokompas account, which means that blinding of the researcher is not possible. Trial participants themselves are also aware of the outcome of the allocation; they receive an e-mail with the outcome of the allocation after they filled in the first questionnaire.

Neither the outcome assessors nor data analysts are blinded regarding the outcome of the allocation. The design of the study is open label; therefore unblinding will not occur. There will be no special criteria for discontinuing or modifying allocated interventions.

### Intervention

Oncokompas is an eHealth self-management application that supports people with cancer and their partners to adopt an active role in the management of their own wellbeing. It supports them in finding and obtaining optimal supportive care, tailored to their own health status, personal characteristics, and preferences. The content of Oncokompas is developed following a stepwise, iterative, and participatory approach, actively involving users and other stakeholders in the design process [30]. In the present study, the version of Oncokompas tailored to partners of patients with incurable cancer is used.

Oncokompas consists of three components: (1) Measure, (2) Learn, and (3) Act. After the log-in procedure, a user enters the first component of Oncokompas which starts with a general questionnaire. Based on this general questionnaire, Oncokompas makes a selection of the topics suitable for this particular user (e.g. when someone has no children, the topic about the relationship with children will not be shown). After this, the user can select which topics he or she wants to address in Oncokompas. The topics target different domains of quality of life; physical, psychological and social functioning, and existential issues. An overview of the topics covered in Oncokompas for partners is shown in Table 2. Subsequently, in the first component “Measure,” a user can complete PROMs on the chosen topics. The PROMs were selected based on Dutch practical guidelines and literature searches, in collaboration with a team of healthcare professionals, partners of patients with cancer, and patients with cancer. Algorithms were developed to link the scores on the PROMs to tailored feedback in the “Learn” component. The algorithms are based on available cut-off scores, Dutch practical guidelines, and/or consensus by teams of experts (i.e. healthcare professionals, partners, and patients).

**Table 2:** Overview of all topics covered in Oncokompas for partners of incurably ill cancer patients

Domain	Topics
Physical	Fatigue Sexuality Sleep problems Shoulder and back pain Changed role of nutrition in the late palliative phase (topic to inform partners)
Psychological	Anxiety (as a result of the patient’s cancer) Coping with emotions Depression Nervousness
Social	Caregiver burden Choices concerning the end-of-life of the patient Loneliness Communication with the physician of the patient Social life Relationship with patient Relationship with children Work issues
Existential	Saying farewell

Then the user enters the “Learn” component, in which feedback on his or her outcomes is provided, tailored to his or her health status, characteristics, and preferences. First, a user gets an overview of his or her overall wellbeing on topic level. A three-color system is used to express the level of wellbeing. When a user is doing well on a topic, he or she gets a green score. An orange score means that a user could use attention and support on that topic. A red score indicates that a user may need professional care. Oncokompas also provides feedback on interrelated symptoms (e.g. caregiver burden and fatigue). The “Learn” component concludes with comprehensive self-care advice, such as tips and tools, tailored to the individual user.

The third step within Oncokompas is the “Act” component, in which users are provided with personalized supportive care options, tailored to their health status and preferences (e.g. preferences for individual therapy versus group therapy). When a user has a red score on a topic, the feedback always includes the advice to contact a healthcare professional, such as a general practitioner or a specialized healthcare professional (e.g. a psychologist) [26]. When a user has an orange score on a topic, the feedback includes suggestions for self-help interventions.

Oncokompas is meant as an additional form of support for partners of patients with incurable cancer. It is not meant as a replacement of a healthcare professional.

### Care as usual

In this study, care as usual is defined as the care provided by any healthcare professional and includes all medical and supportive care that partners of patients with incurable cancer would receive, regardless of their participation in this study.

### Outcome assessment

Caregiver burden is the primary outcome measure used to assess the efficacy of Oncokompas among partners of patients with incurable cancer. Secondary outcome measures are general self-efficacy and health-related quality of life. In addition, outcomes on cost-utility will be measured.

Measurements will be collected at baseline (t0), two weeks after randomization (t1), and three months after the baseline measurement (t2). Measurements will be assessed through online questionnaires. An overview of the primary and secondary outcome measures is shown in Table 3.

**Table 3:** Measurement overview

Aim		Outcome measures	Instrument
Efficacy	Primary outcome measure Secondary outcome measures	Caregiver Burden Self-efficacy	Caregiver Strain Index + (CSI+) General Self-Efficacy Scale (GSE)
Cost-utility		Health-related quality of life Medical costs Productivity costs Costs of Informal Care	EuroQol 5 Dimensions (EQ-5D-5L) iMTA Medical Consumption Questionnaire (iMCQ) iMTA Productivity Cost Questionnaire (iPCQ) iMTA Valuation of Informal Care Questionnaire (iVICQ)

### Primary outcome measure

#### Caregiver strain index +

Caregiver burden is assessed with the Caregiver Strain Index + (CSI+). The CSI+ is an extended version of the Caregiver Strain Index, developed in 1983 [31]. The original 13-item CSI measures the burden that informal caregivers experience as a result of caring for their loved ones. In the CSI+ questionnaire, five positive items were added to the original CSI. These positive items fall into two categories: “coping” factors and “attitudinal” factors. All items of the CSI+ are completed with “yes” or “no” and are equally weighted to calculate a carer’s total CSI+ score. Research showed that the internal consistency (Cronbach’s alpha) for the 13-item CSI was 0.86 [31]. Furthermore, a study testing the feasibility and validity of the CSI+ reported that by including positive aspects of care, resulting in the CSI+, an improved convergent validity of the Caregiver Strain Index is realized [32].

### Secondary outcome measures

#### General self-efficacy scale

The General Self-Efficacy Scale (GSE) is a 10-item questionnaire, assessing how a person deals with difficult situations in his or her life. The items have a 4-point Likert scale, in the range of 1–4 (i.e. not at all true, hardly true, moderately true, and exactly true). The total score is in the range of 10–40 and is calculated by adding up the scores on the 10 items. A higher score indicates a greater generalized sense of self-efficacy [33]. A study examining the psychometric properties of the GSE showed that the GSE scale is reliable, homogeneous, and unidimensional [34].

### Cost-utility evaluation

To evaluate the cost-effectiveness of Oncokompas compared to current care, a cost-utility analysis will be conducted in which the difference in total three-months costs between the two study arms is compared to the difference in quality-adjusted life years (QALYs) based on the EuroQol 5 Dimensions.

#### Health-related quality of life

The EuroQol 5 Dimensions (EQ-5D-5L) will be used to measure health-related quality of life on five dimensions of health (i.e. mobility, self-care, usual activities, pain/discomfort, and anxiety/depression), presented to the respondent by five items which all have five answer categories (i.e. no problems, some problems, moderate problems, severe problems, and extreme problems/unable to). As a result, the EQ-5D-5L can describe 3125 unique health states. After completion of the questionnaire, the profile of answers can be transformed to a value given by the general public using the Dutch index tariff of the EQ-5D index [35]. The EQ-5D is a validated questionnaire to measure health-related quality of life [36, 37].

#### Medical consumption questionnaire, productivity cost questionnaire, and valuation of informal care questionnaire

To measure the costs of healthcare, the costs for patients and their families (e.g. travelling costs and help received from family and friends), and costs within other sectors (i.e. productivity losses from paid and unpaid work) in the previous three months, an adapted version of the medical consumption questionnaire (iMCQ) and productivity cost questionnaire (iPCQ) will be used. An adapted version of the valuation of informal care questionnaire (iVICQ) will be used for the valuation of informal care by monetary and non-monetary methods. All these questionnaires are developed by the Institute for Medical Technology Assessment of the Erasmus University Rotterdam (iMTA), the Netherlands [38–40].

#### Sociodemographic characteristics and health-related characteristics

A study-specific questionnaire will be used at baseline (t0) to assess the sociodemographic characteristics (e.g. age, education level, and work situation) and health-related characteristics of the partner as well as the health situation of the patient with cancer.

### Sample size

To demonstrate the presence of an effect on the CSI+ of at least 0.5 standard deviations as statistically significant in a one-tailed test at alpha = 0.05 and a power of (1 - beta) = 0.80, a minimum of 51 participants in each condition of the RCT will be required at follow-up. Anticipating a drop-out rate of 25% between t0 and t2, 68 participants per condition need to be included at t0. Therefore, the total study cohort comprises 136 partners of patients with incurable cancer.

### Statistical analyses

All analyses will be conducted in agreement with the intention-to-treat principle. Descriptive statistics will be used to describe the sociodemographic characteristics, health-related characteristics of the partner, the health situation of the patient with cancer, and the outcome measures. Chi-square tests and independent samples t-tests will be used to analyze whether randomization resulted in a balanced distribution of sociodemographic and health-related characteristics across the study arms. Mann–Whitney U tests will be performed in case of non-normality of the data. To test whether there are differences in the outcomes across the study arms at baseline, independent samples t-tests will be used.

Linear mixed models (LMM) will be used to determine the efficacy of Oncokompas by comparing longitudinal changes between the intervention group and control group with fixed effects for study arm, time, and their two-way interaction, as well as a random intercept for individuals.

LMM will also be used to determine whether age, gender, socioeconomic status (e.g. education level and work situation), the health situation of the patient, and baseline quality of life moderate the efficacy of Oncokompas with fixed effects for study arm, time, the potential moderator, and all two-way and three-way interaction effects, as well as a random intercept for individuals.

Post-hoc analyses will be applied when significant results are found in the efficacy and moderation analyses. To measure the differences in change between the intervention group and control group at follow-up measurements, independent samples t-tests with Bonferroni correction will be used. These tests will also be used to assess whether change scores between the intervention group and control group differed significantly within each category of the significant moderator variables.

The effect sizes (ES) of the intervention will be measured by calculating the (between group) Cohen’s d. The magnitude of the ES is classified as large (≥ 0.80), moderate (0.50–0.79) or small (< 0.50) [41].

A *p* value < 0.05 will be considered significant for all analyses. All tests will be one-tailed. IBM Statistical Package for the Social Sciences (SPSS) version 26 (IBM Corp., Armonk, NY USA) will be used to perform all statistical analyses.

#### Economic outcomes

The analysis of economic outcomes will also be conducted in agreement with the intention-to-treat principle. An incremental cost-utility ratio (ICUR) will be calculated by dividing the incremental costs (i.e. mean costs in the intervention group minus mean costs in the control group) by the incremental QALYs (i.e. mean QALYs in the intervention group minus mean QALYs in the control group).

Total costs from a societal perspective will be calculated using intervention costs, costs of healthcare (i.e. costs of healthcare and medication), costs for patients and their families (e.g. travelling costs and help received from family and friends), and costs within other sectors (i.e. productivity losses from paid and unpaid work). Intervention costs and costs of healthcare will be used to calculate total costs from a healthcare perspective.

Costs of healthcare and costs for patients and their families will be calculated by multiplying resource use by integral costs prices as presented in the Dutch Health Care Insurance Board (CVZ) guidelines on cost studies [42]. The friction cost method will be used to calculate the costs within other sectors [43, 44].

The time horizon will be set at three months follow-up; therefore, neither costs nor effects will be discounted. The EQ-5D utility score will be used to calculate QALYs by linking the scores to the various health states of the EQ-5D. Multiple imputation will be used when data are missing on the costs of healthcare, the costs of patients and their families, and the costs within other sectors. This also accounts for missing data on the utilities measured with the EQ-5D.

To obtain 95% confidence intervals around the costs and QALY differences, non-parametric bootstrapping with 5000 imputations will be used. A cost-utility plane will be plotted for the projection of the resulting pairs of cost and effect differences. A cost-effectiveness acceptability curve will be made to reflect the probability of Oncokompas being cost-effective given different willingness-to-pay ceilings [45]. Sensitivity analyses will be conducted focusing on uncertainty in the main cost factors.

### Monitoring

Since this trial concerns a low-risk intervention (i.e. access to an online application), no independent Data Monitoring Committee is required for this study. The research team will meet monthly to discuss all study activities (i.e. the daily management and organization of the study, such as the recruitment of participants and participant monitoring) and feasibility of the study (i.e. time management of the trial).

## Discussion

This study, targeting partners of patients with incurable cancer, will assess the efficacy of Oncokompas as an eHealth self-management application on caregiver burden, self-efficacy, and quality of life, and its cost-utility from a healthcare and societal perspective, compared to care as usual.

Partners of patients with incurable cancer often face challenges due to the patient’s diagnosis and cancer treatment. These challenges, such as emotional and financial difficulties, influence their daily lives and health. Partners are often involved in the illness trajectory by providing physical, emotional, and practical assistance to the patient [1, 46]. Although there are positive aspects related to informal caregiving (e.g. feeling rewarded or experiencing a sense of personal growth) [8], partners often also feel distressed and burdened due to their caregiving responsibilities [10].

A meta-analysis, investigating different types of interventions offered to family caregivers of cancer patients, showed that interventions targeting caregivers alone have better outcomes regarding caregivers’ perceptions of their caregiving experiences than interventions provided to cancer patients and their caregivers jointly [47]; these targeted interventions are better able to focus on the needs of the caregivers.

Given et al. reported that informal caregivers often are gatekeepers to themselves; they may hesitate to seek help for their own needs [10], for example because they protect the patient from their own complaints or because they do not want to shift the attention from the patient to themselves [16]. It might be hard for partners to discuss certain issues with healthcare professionals (e.g. their fears about losing the patient, the strain they experience because their partner has cancer, or their sexual needs), especially in presence of the patient. By informing partners and providing self-care advice on a wide variety of symptoms which could possibly affect their quality of life, Oncokompas could be a solution to meet unmet needs of partners of patients with incurable cancer. Furthermore, Oncokompas could stimulate partners and patients to talk about the patient’s and partner’s wishes regarding the end-of-life phase of cancer. Oncokompas can be used by partners at their own time in their own home. This is an advantage, because partners often are already burdened due to the patient’s cancer. To use Oncokompas, partners do not have to take time off from work or find respite care for the patient.

In a study investigating the preferences and attitudes regarding Oncokompas as a system monitoring symptoms, it was reported that caregivers of patients with glioma expected that Oncokompas could decrease the barriers to contact healthcare professionals for their own needs [48]. Köhle et al. found that partners of patients with cancer are interested in using web-based supportive interventions and would be interested in obtaining online information when they know the patient has an incurable disease. Other topics of interest identified in that study were how partners could take care of themselves and how they could cope with emotions [49]. Previous studies indicated that palliative care interventions may improve quality of life among caregivers of patients with advanced cancer [19, 47, 50]. It has also been suggested that interventions targeting caregivers may also have a positive impact on a patient’s symptoms [50]. This is worth noting, since research has shown that the level of distress in the informal caregiver is also related to the wellbeing of the patient, for example the severity of their symptoms and their level of functional autonomy [51, 52].

Since caregiver burden could lead to a deterioration in quality of life, reductions in work productivity, and an increase in the use of healthcare resources [53], it is important to investigate the costs and effects related to caregiving, while investigating the effects of interventions [54]. In this study, medical costs, productivity costs, and costs of informal caregiving will be taken into account in the cost-utility analysis. It is expected that Oncokompas will improve QALYs at acceptable costs, compared to care as usual. This study will create knowledge on the impact of informal cancer care, which in its turn could serve as valuable information for policy makers to take into account while developing healthcare arrangements regarding the facilitation and support of informal caregiving.

This study will also contribute to the knowledge about the effectiveness of eHealth interventions used by partners of patients with incurable cancer. When Oncokompas is proven to be effective for partners, this may stimulate the implementation of the intervention as a health service for partners of cancer patients.

## Trial status

The recruitment of participants for this study started in March 2019 and is still ongoing. The recruitment is expected to be complete in March 2020. The results of this study have not been published in any publications and have not been submitted to any other journal.

## Supplementary information

**Additional file 1.** SPIRIT Checklist

## Data Availability

The datasets analyzed during the current study will be available in the EASY repository of DANS-KNAW after completion of the thesis that will be written reserving the generated data. The (intellectual) property rights with regard to the generated data will reside at the Vrije Universiteit Amsterdam. Interested parties can request a non-exclusive license for research and educational purposes. The non-exclusive license may be requested only after the completion of the thesis that will be written reserving the generated data.

## Abbreviations

CSI: Caregiver Strain Index
CVZ: Dutch Health Care Insurance Board
EQ -5D: EuroQol 5-Dimensions questionnaire
ES: Effect size
GSE: General self-efficacy
HRQOL: Health-related quality of life
ICUR: Incremental cost-utility ratio
iMCQ: iMTA Medical Consumption Questionnaire
iMTA: Institute for Medical Technology Assessment
iPCQ: iMTA Productivity Cost Questionnaire
iVICQ: iMTA Valuation of Informal Care Questionnaire
LMM: Linear mixed models
METc: Medical Ethical Committee
PROMs: Patient-reported outcome measures
QALY: Quality-adjusted life year
RCT: Randomized controlled trial
SPIRIT: Standard Protocol Items: Recommendations for Interventional Trials
